# Malaria and curable sexually transmitted and reproductive tract coinfection among pregnant women in rural Burkina Faso

**DOI:** 10.1186/s41182-021-00381-5

**Published:** 2021-11-04

**Authors:** Moussa Lingani, Serge H. Zango, Innocent Valéa, Massa dit A. Bonko, Sékou O. Samadoulougou, Toussaint Rouamba, Marc C. Tahita, Maïmouna Sanou, Annie Robert, Halidou Tinto, Philippe Donnen, Michèle Dramaix

**Affiliations:** 1 École de Santé Publique, Université Libre de Bruxelles, Bruxelles, Belgique; 2 Institut de Recherche en Sciences de la Santé/Direction Régionale du Centre Ouest (IRSS/DRCO), Nanoro, Burkina Faso; 3 Epidemiology and Biostatistics Research Division, Institut de Recherche Expérimentale et Clinique, Université Catholique de Louvain, Bruxelles, Belgique; 4 Evaluation Platform On Obesity Prevention, Quebec Heart and Lung Institute Research Center, Quebec, Canada

**Keywords:** Pregnancy, Malaria, Syphilis, Chlamydia, Bacterial vaginosis, Coinfection, Burkina Faso

## Abstract

**Background:**

Malaria and sexually transmitted/reproductive tract infections (STI/RTI) are leading and preventable causes of low birthweight in sub-Saharan Africa. Reducing their impact on pregnancy outcomes requires efficient interventions that can be easily integrated into the antenatal care package. The paucity of data on malaria and STI/RTI coinfection, however, limits efforts to control these infections. This study aimed to determine the prevalence and associated factors of malaria and STI/RTI coinfection among pregnant women in rural Burkina Faso.

**Methods:**

A cross-sectional survey was conducted among 402 pregnant women attending antenatal clinics at the Yako health district. Sociodemographic and behavioral data were collected, and pregnant women were tested for peripheral malaria by microscopy. Hemoglobin levels were also measured by spectrophotometry and curable bacterial STI/RTI were tested on cervico-vaginal swabs using rapid diagnostic test for chlamydia and syphilis, and Gram staining for bacterial vaginosis. A multivariate logistic regression model was used to assess the association of malaria and STI/RTI coinfection with the characteristics of included pregnant women.

**Results:**

The prevalence of malaria and at least one STI/RTI coinfection was 12.9% (95% confidence interval, CI: [9.8–16.7]), malaria and bacterial vaginosis coinfection was 12.2% (95% CI: [9.3–15.9]), malaria and chlamydial coinfection was 1.6% (95% CI: [0.6–3.8]). No coinfection was reported for malaria and syphilis. The individual prevalence was 17.2%, 7.2%, 0.6%, 67.7% and 73.3%, respectively, for malaria infection, chlamydia, syphilis, bacterial vaginosis and STI/RTI combination. Only 10% of coinfections were symptomatic, and thus, 90% of women with coinfection would have been missed by the symptoms-based diagnostic approach. In the multivariate analysis, the first pregnancy (aOR = 2.4 [95% CI: 1.2–4.7]) was the only factor significantly associated with malaria and STI/RTI coinfection. Clinical symptoms were not associated with malaria and STI/RTI coinfection.

**Conclusion:**

The prevalence of malaria and curable STI/RTI coinfection was high among pregnant women. The poor performance of the clinical symptoms to predict coinfection suggests that alternative interventions are needed.

## Introduction

In sub-Saharan Africa (SSA), 880,000 stillbirths and 1.2 million neonatal deaths occur each year [1, 2]. Low birthweight, (LBW, a birthweight < 2500 g), from intrauterine growth retardation, preterm birth, or both is the leading cause of neonatal death [3]. Intrauterine infections such as malaria and sexually transmitted/reproductive tract infections (STI/RTI) are implicated in the occurrence of LBW [4]. For instance, while malaria infections highly predispose the fetus to intrauterine growth retardation [5], untreated STI /RTI is associated to prematurity [6]. A number of studies have assessed the magnitude of these infections in SSA. Indeed, in a review conducted in west African pregnant women, the prevalence of malaria infection was 32%, syphilis was 3.5%, bacterial vaginosis (BV) was 37.6%, and chlamydial infection was 6.1%, but the study failed to report on malaria and STI/RTI coinfection [7]. Another study estimated that 38% of Zambian pregnant women were coinfected with malaria and at least one STI/RTI [8]. Pregnant women, despite their high susceptibility to infections in SSA are usually asymptomatic, which severely limits clinical detection and appropriate case management [9–11].

Antenatal care (ANC) is a unique opportunity to screen, treat and prevent health conditions that negatively affect the pregnancy outcomes. The intermittent preventive treatment of pregnant women using sulfadoxine–pyrimethamine (IPTp-SP) is recommended to prevent malaria infection and its associated negative effects on the pregnancy outcome [12]. In the meantime, STI/RTI management relies on adequate diagnosis and treatment. While for syphilis, there is a simple and easy to use rapid diagnostic test available, such tools are not available for chlamydial infection nor for bacterial vaginosis. In addition, the limited laboratory resources for etiological diagnosis in SSA [13], have led to the development of a syndromic approach [14], in which a group of symptoms are identified and efficacious treatments provided by following a simplified algorithm [15].

In Burkina Faso, the prevalence of malaria infected pregnant women during the peak transmission varies between 20 and 50% in the rural area depending on the climatic patterns [11, 16]. Despite that STI/RTI risk to the mother and her offspring is well established, their prevalence remains poorly documented. In a study conducted among pregnant women in Burkina Faso, the prevalence of syphilis was 1.7% and that of bacterial vaginosis was 6.4% [17, 18]. Another study reported a prevalence of 13% for bacterial vaginosis, 3.6% for syphilis, 3.1% for chlamydial infection, 3% for genital warts, 1.6% for gonococcal infection, 0.6% for genital ulcer, and 32.4% for at least one STI/RTI [19]. A recent evaluation which used a syndromic approach for diagnostic reported a prevalence of 4.0% for STI/RTI [20]. The only study that assessed malaria and STI/RTI coinfection reported a prevalence of 1.2% in two rural health districts of the country [20]. This paucity of data on malaria and STI/RTI coinfections in pregnancy has prevented the development of adequate and integrated interventions to control their associated adverse effects on the pregnancy. Thus, adequately quantifying the magnitude of malaria and STI/RTI coinfection among pregnant women is needed to inform policymakers and update antenatal care package accordingly. This study aimed to determine the prevalence and associated factors of malaria and STI/RTI coinfection among pregnant women at their first antenatal care visit in rural Burkina Faso.

## Methods

### Study area

The study was conducted in the antenatal clinics of three peripheral health centers (district 4, district 5 and district 6) of the Yako health district, northern part of Burkina Faso. In this area, malaria transmission is holo-endemic with a peak of transmission during the rainy season (July–November) [21]. The health district catchment area covers 424,577 inhabitants, and 23,000 pregnancies were recorded in 2017 [21]. In 2017, malaria was the main reason of consultation, hospitalization and death which particularly affected children under 5 years of age and pregnant women [21].

### Study design

This was an ancillary study of a main project comparing the efficacy of IPTp-SP versus IPTp-SP plus azithromycin to prevent low birthweight (www.pactr.org, registration number PACTR201808177464681). At enrollment, pregnant women were screened for malaria and STI/RTI (syphilis, chlamydia and bacterial vaginosis) between August 2019 and September 2020.

### Inclusion/exclusion criteria

Eligibility criteria included age between 16 and 45 years, gestation ≤ 24 weeks, main residence in the health district, willingness to deliver in the study area and written informed consent. Exclusion criteria included the inability to adhere to the study procedures, or any uptake of an antimalarial or an antibiotic drug within the last month preceding the selection visit.

### Data collection procedures

At enrolment, pregnant women that provided a written informed consent were examined to collect socio-demographic, obstetric, medical characteristics and malaria prevention measures. Malaria infection and bacterial STI/RTI were tested from peripheral blood smears and cervico-vaginal swabs. We used the one step anti—*Treponema pallidum* rapid diagnostic test SD-Bioline Syphilis 3.0 (sensitivity 99.3% and specificity of 99.5%) (Standard Diagnostics Inc. Korea) for syphilis test on blood samples, and the one-step chlamydia antigen rapid Test SD-Bioline Chlamydia (sensitivity 93.1% and specificity of 98.8%) (Standard Diagnostics Inc. Korea) for chlamydia detection on cervical swabs. Hemoglobin level was measured using a portable spectrophotometer (HemoCue, Ängelholm, Sweden). The antenatal clinics provided HIV counselling and testing; however, these data were not included in this analysis as the approved study protocol did not include HIV data collection. Malaria was tested by standard microscopy at the laboratory of the Clinical Research Unit of Nanoro (CRUN) and bacterial vaginosis (BV) tested by Gram staining and the determination of the Nugent score [22].

### Biological sample collection and laboratory procedures

For malaria infection, a nurse collected peripheral blood samples by finger pricks, prepared thick and thin blood smears for standard microscopy. The slides were stained with 5% Giemsa for 30 min and independently double examined by two certified microscopists*.* In case of discrepancy (discrepant species or count difference of at least 50%), a third independent reading was done. The final result being the average of the two closest results.

For bacterial vaginosis, vaginal swabs were collected by trained midwives using dry sterile cotton swabs (Heinz Herenz Medical, Hamburg, Germany), which were transferred within an hour to the microbiology laboratory for analysis. Once at the laboratory, macroscopic, microscopy examinations and Gram staining were performed by trained technicians. The slides were stained and examined by microscopy at *100-x* to identify different bacterial forms to established the Nugent score. This score was calculated by assessing the presence of large bacteria Gram-positive rods (*Lactobacillus* morphotypes; scored from 0 to 4), small Gram-variable rods (*Gardnerella vaginalis* morphotypes; scored from 0 to 4), and curved Gram-variable rods (*Mobiluncus spp* morphotypes; scored from 0 to 2) [22]. We calculated the Nugent score by summing the three sub-scores together (*Lactobacillus* species; *Gardnerella vaginalis*; and *Mobiluncus spp*). A score greater or equal to 7 was considered a bacterial vaginosis [22].

For chlamydial infection, trained midwives used non-lubricated specula and sterile swabs provided with the SD-Bioline chlamydial diagnosis kit to collect the samples. A first sterile swab was used to collect the excess mucus at the exocervix level which was discarded, then a second swab was used to collect the sample in the endocervical canal. The swabs were transferred to the laboratory of microbiology, where *Chlamydia Trachomatis* presence was detected by chromatography according to the rapid test kit manufacturer’s instructions.

### Sample size calculation

In SSA, the prevalence of malaria and at least one STI/RTI coinfection among pregnant women is estimated around 38% in 2016 [8]. With the hypothesis that this prevalence would be similar in Burkina Faso, the required sample size was calculated using the Cochran formula *n* = Z^2^ *p*(1-*p*)/*i*^2^, where *p* = 38% is the expected proportion, *i* = 5%, the margin of error, and *Z* corresponds to the 95% confidence interval (1.96). A minimum of 362 participants were required.

### Data processing and analysis

Data was collected on a paper-based questionnaire, subsequently double entered onto OpenClinica software, and exported onto RStudio (Version 1.2.5042) for cleaning and analysis. We designed a four-level variable to group women according to the number of infections: (1) malaria and STI/RTI coinfection; (2) malaria but no STI/RTI; (3) only STI/RTI; and (4) no infection. We dichotomized gravidity variable as primigravidae (one pregnancy) and multigravida (2 or more pregnancies), and we used frequency tables to summarize categorical variables. We used mean or median with respective standard deviations or quartiles for numerical variables. To investigate factors associated with coinfection, we conducted a univariate logistic analysis to calculate odds ratios (OR) and their 95% confidence intervals (95% CI). We subsequently selected all factors with a tendency for association in the univariate analysis (*p* < 0.1) and used them as a starting model for a backward stepwise elimination process and keeping variable with a *p* value < 0.1 in the final model. Women age was excluded from the multivariate analysis due to a strong correlation with gravidity. The significance level used was set at 5% (two-sided *p* value).

## Results

### Sociodemographic, obstetric and behavioral characteristics

**Fig. 1:**
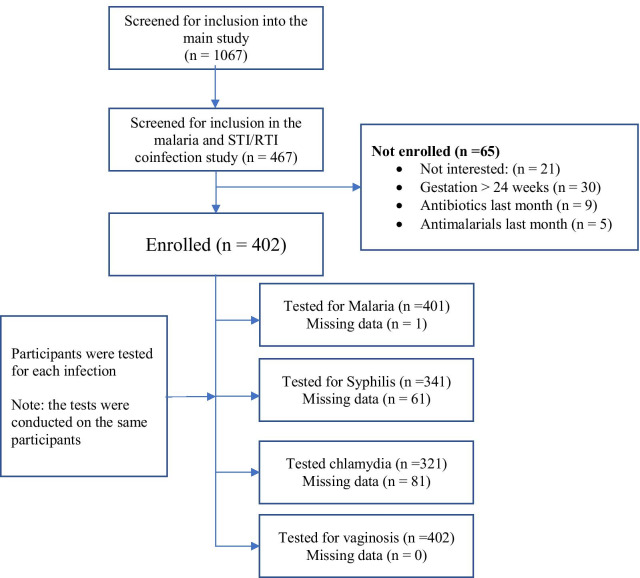
Pregnant women participants’ flow chart. STI/ RTI: sexually transmitted /reproductive tract infection

A total of 1067 first antenatal care attendees were screened to enroll 972 pregnant women in the main study, but only 467 from three antenatal clinics were screened to enroll 402 participants in the ancillary study (Fig. 1). Therefore, all analyses in this manuscript are based on the 402 pregnant women from three peripheral health centers: 325 (80.8%) from the district N°4, 47 (11.7%) from the district N°5 and 30 (7.5%) from the district N°6. The baseline characteristics of study participants are summarized in Table 1.

**Table 1:** Baseline characteristics of pregnant women at their first ANC visits in the Yako Health District, Burkina Faso (*n* = 402)

Characteristics	*n* (%)
Age group (in years)	
≤ 20	99 (24.7)
21–25	122 (30.4)
26–30	75 (18.7)
> 30	105 (26.2)
Missing*	1
Marital status	
Single	19 (4.7)
Married or living together	383 (95.3)
Level of education	
With formal education	235 (58.5)
Without formal education	167 (41.5)
Occupation	
Employed/self employed	87 (21.7)
Unemployed	223 (55.6)
Students	91 (22.7)
Missing*	1
Alcohol use (> one standard drink/day)	
Yes	73 (18.2)
No	329 (81.8)
ITN use	
Yes	312 (77.6)
No	90 (22.4)
Gravidity	
Primigravidae (first pregnancy)	121 (30.1)
Multigravida (≥ 2 pregnancies)	281 (69.9)
History of stillbirth or abortion	
Yes	62 (15.4)
No	340 (84.6)
Condom at last sex	
Yes	20 (5.0)
No	377 (95.0)
Missing*	5
Age at first sex (in years)	
< 20	277 (68.9)
≥ 20	125 (31.1)
Body mass index (kg/m^2^)	
< 18.5	16 (4.0)
18–25	288 (72.2)
> 25	95 (23.8)
Missing*	3
At least one clinical symptom**	
No	364 (90.5)
Yes	38 (9.5)
Hemoglobin level (g/dL)	
≥ 11	126 (36.5)
< 11	219 (63.5)
Missing*	57

*ITN* long lasting insecticide treated bed net, *g/dL* gram per deciliter, *ANC* antenatal care

*Missing values were not included in calculations, but only presented as number

**Clinical symptoms included fever, vaginal discharge, vaginal burn, vulvar or perianal itching and pelvic pain

The median age of participants was 24 years (IQR [interquartile range]: 21–30). Nearly all women had partners (married or living together) at the time of the survey (95.3%) and 55.6% of them were unemployed (housewives). The overall highest educational level was low as nearly four in ten women (41.5%) had never been at school and only 2.2% reached university level. The body mass index (BMI) was normal (from 18.5 to 25 kg per m^2^) in the majority of women (72.2%), whereas one quarter (23.8%) were overweighed and only 4.0% were under-weighed.

Nearly one-tenth (9.5%) of women had at least one clinical symptom such as fever (axillary temperature ≥ 37.5 degree centigrade), vaginal burn, pelvic pain, vaginal discharge, or vulvar/perianal itching at the clinical and obstetrical examination. The most common symptom was vaginal itching (20/402), and followed by vaginal discharge (18/402). A high proportion (63.5%) of pregnant women were also anemic (4.6% had severe anemia, whereas 58.8% had moderate anemia).

Most of the pregnant women declared to have their first sexual intercourse before their 20th birthday (68.9%) and only a minority of them (5%) confirmed the use of condom during their last sexual intercourse. Alcohol use defined as a daily uptake of more than one “standard drink” (roughly containing 10 g of pure alcohol) was common (18.2%). Nearly three-quarter (77.6%) of women declared the use of insecticide treated bed net (ITN) the night before their visit.

One third of women (30.1%) visited the health center for their first pregnancy, whereas the other two-third (69.9%) visited for their second or subsequent pregnancies. Miscarriage and stillbirth were frequent in the study setting (15.4%).

### Prevalence of infections

The prevalence of malaria infections was 17.2% (95% confidence interval (CI): [13.7–21.3]) and all were due to the *Plasmodium falciparum* species (Table 2). Regarding individual STI/RTI, the prevalence was 7.2% (95% CI: [4.7–10.7]) for Chlamydia, 0.6% (95% CI: [0.1–2.3]) for syphilis, and 67.7% (95% CI: [62.8–72.2]) for bacterial vaginosis. The proportion of women tested positive for at least one STI/RTI was 73.3% (95% CI: [68.5–77.6]) (Table 2).

**Table 2:** Prevalence of malaria and sexually transmitted infections among first ANC attendees of the Yako Health District, Burkina Faso, *n* = 402

Infection	Categories	*n*	Percentage [95% CI]
Malaria infection	Positive	69	17.2 [13.7–21.3]
	Negative	332	82.8 [78.6–86.3]
	Missing*	1	–
Chlamydial infection	Positive	23	7.2 [4.7–10.7]
	Negative	298	92.8 [89.3–95.3]
	Missing*	81	–
Syphilis	Positive	2	0.6 [0.1–2.3]
	Negative	339	99.4 [97.7–99.9]
	Missing*	61	–
Bacterial vaginosis	No	130	32.3 [27.8–37.2]
	Yes	272	67.7 [62.8–72.2]
At least one STI/RTI	Yes	280	73.3 [68.5–77.6]
	No	102	26.7 [22.4–31.5]
	Missing*	20	–

*CI* confidence interval, *STI/RTI* sexually transmitted infection/reproductive tract infection, *ANC* antenatal care

*Missing values were not included in calculations, but only presented as number

The prevalence of malaria and at least one STI/RTI coinfection was 12.9% (95% CI: [9.8–16.7]) and that of malaria and bacterial vaginosis coinfection was 12.2% (95% CI: [9.3–15.9]). Cases of syphilis were detected but no coinfection with malaria was reported (Table 3).

**Table 3:** Prevalence of malaria and curable STI/RTI coinfection among first antenatal care attendees in the Yako health district, Burkina Faso (*n* = 402)

Coinfection	Categories	Count	Percentage (%)	[95% CI]
Malaria infection and all STI/RTI	Malaria and at least on STI/RTI	49	12.9	[9.8–16.7]
	Malaria only	15	3.9	[2.3–6.6]
	STI/RTI only	230	60.4	[55.2–65.3]
	No infection	87	22.8	[18.8–27.4]
Malaria infection and bacterial vaginosis	Yes	49	12.2	[9.3–15.9]
	Missing*	1	–	–
Malaria infection and Chlamydia	Yes	5	1.6	[0.6–3.8]
	Missing*	1	–	–
Malaria infection and syphilis	Yes	0	0	[0.0–1.4]
	Missing*	62		–

*CI* confidence interval, *STI/RTI* sexually transmitted infection/reproductive tract infection

*Missing values were not included in calculations, but only presented as number

### Factors associated with malaria and at least one STI/RTI coinfection

In the univariate analysis, women under 20 years of age, and women at their first pregnancy were at significantly higher risk of coinfection (*p* value = 0.01). Anemic women (OR = 2.0 [95% CI: 1.0–4.5], *p* value = 0.06) and unemployed women (OR = 2.1 [95% CI: 0.9–5.7], *p* value = 0.1) were also at increased risk of coinfection but did not reach significance level. In the final multivariate model, malaria and at least one STI/RTI coinfection was significantly associated with the first pregnancy (aOR = 2.4 [1.2–4.7], *p* value = 0.01) (Table 4). Unemployment (aOR = 2.2 [0.9–6.0], *p* value = 0.08) and anemia (aOR = 1.8 [0.8–4.1], *p* value = 0.1) tended to be associated with malaria and STI/RTI coinfections but without reaching statistically significance level (Table 4). Clinical symptoms were not associated with malaria and STI/RTI coinfection (0.8 [0.2–2.2], *p* value = 0.7).

**Table 4:** Univariate and multivariate analyses of potential risk factors for malaria and curable STI/RTI coinfection among first ANC attendees (*n* = 402) in the Yako health district, rural Burkina Faso

Characteristics	*n* (%)	^1^Positive *n* (%)	OR [95% CI]	aOR [95% CI]
Age group (in years)				
≤ 20	98	20 (20.4)	**2.6 [1.2–6.4]	–
21–25	121	14 (11.6)	1.3 [0.6–3.3]	
26–30	75	6 (8.0)	0.9 [0.3–2.6]	
> 30	101	9 (8.9)	Ref	
Marital status				
Single	19	2 (10.5)	Ref	–
Married	377	47 (12.5)	1.2 [0.3–7.8]	
Level of education				
With formal education	234	31 (13.2)	1.22 [0.7–2.3]	–
Without formal education	162	18 (11.1)	Ref	
Occupation				
Employed/self employed	87	6 (6.9)	Ref	Ref
Unemployed	217	29 (13.4)	2.1 [0.9–5.7]	*2.2 [0.9–6.0]
Students	91	14 (15.4)	*2.4 [0.9–7.2]	1.6 [0.6–4.9]
Alcohol use				
Yes	71	6 (8.5)	0.6 [0.2–1.4]	–
No	325	43 (13.2)	Ref	
ITN use				
Yes	307	37 (12.1)	Ref	–
No	89	12 (13.5)	1.1 [0.5–2.2]	
Gravidity				
Primigravidae (1)	121	23 (19.0)	**2.2 [1.2–4.1]	**2.4[1.2–4.7]
Multigravida (≥ 2)	275	26 (9.5)	Ref	Ref
Condom at last sex				
Yes	19	2 (10.5)	Ref	–
No	372	46 (12.4)	1.2 [0.3–7.7]	
Age at first sex (in years)				
< 20	271	36 (13.3)	1.3 [0.7–2.7]	–
≥ 20	125	13 (10.4)	Ref	
Body mass index (kg/m^2^)				
Normal (≥ 18.5 & < 25)	284	34 (12.0)	Ref	–
Abnormal (< 18.5 or ≥ 25)	15	1 (6.7)	0.5 [0.1–2.7]	
At least one clinical symptom				
No	359	45 (12.5)	Ref	
Yes	37	4 (10.8)	0.8 [0.2–2.2]	
Hemoglobin level (g/dL)				
≥ 11	123	10 (8.1)	Ref	Ref
< 11	216	33 (15.3)	*2.0 [1.0–4.5]	*1.8 [0.8–4.1]

*p value < 0.1, **p value < 0.05, ^1^malaria and at least one STI/RTI coinfection

*CI* confidence interval, *STI/RTI* sexually transmitted infection/reproductive tract infection, *OR* odds-ratio, *aOR* adjusted odds-ratio, *ITN* long lasting insecticide treated bed net, *g /dL* gram per deciliter, *ANC* antenatal care

*Missing values were not included in calculations, but only presented as number

^1^Variable gravidity, occupation, and anemia were included in the multivariate logistic regression model

## Discussion

In our study nearly 13% of pregnant women were coinfected with malaria and at least one STI/RTI, and this prevalence increased to 20% at first pregnancy, which emphasized the high burden of these infections in the study population. So far, no other study in Burkina Faso has assessed the coinfection of malaria and STI/RTI using a prospective approach, and this could be the reason why malaria and STI/RTI coinfection has long been neglected as a public health problem. Indeed, the only study which reported a prevalence of 1.2% coinfections was a retrospective study on data collected through the routine system [20]. The relatively low prevalence of malaria and STI/RTI coinfection in that study could be explained by the low prevalence of STI/RTI (4.0%) detected through the symptom-based approach [20]. It is very likely that the symptom based approach grossly underestimated the actual prevalence as several studies have demonstrated the poor performance of the syndrome-based diagnosis of STI/RTI in pregnancy [9, 23, 24].

Bacterial vaginosis and malaria had the highest coinfection rate in this study. The high prevalence of bacterial vaginosis in this study (67.7%) increased the risk of coinfection. Indeed, previous study conducted in the country reported much lower rates of bacterial vaginoses with 6.4% in 2008 [18], and 12.2% in 1997 [19]. Although the prevalence of bacterial vaginosis is well described in the study setting, little evidence supports the benefit of antibacterial treatment to improve pregnancy outcomes [25].

We did not report any coinfection of malaria and syphilis although, separately, both infections were reported. The low prevalence of syphilis reported in this study (0.6%) is likely the reason behind this absence of coinfection between the two infections. However, the low prevalence of syphilis and the absence of coinfection in this study should not undermine the systematic screening of these infections as the cost effectiveness of such intervention is well established even in a context, where the rate is lower than 2% [26, 27]. In addition, the high burden of syphilis negative impact on pregnancy outcome reported in Tanzania (up to 24% preterm birth) stays as a reminder of its importance and also suggests the need for efficient interventions [28]. The systematic screening and penicillin treatment of syphilis in Burkina Faso since 2014 may have reduced its prevalence and thus its coinfection with malaria in the current study. Indeed, whereas Meda et al. reported 3.1% in 1997 [19], Kirakoya-Samadoulougou et al. reported half of this prevalence 20 years later (1.7%) [17].

We reported a low proportion of pregnant women coinfected with malaria and chlamydial coinfection in the study settings. The use of rapid diagnosis test in this study likely understated the actual prevalence of chlamydial infections and thus its coinfection with malaria. Indeed, PCR-based diagnostic demonstrated higher prevalence in East Africa with 13% of women infected with *Chlamydia Trachomatis* [8, 29]. Given the neonatal complications associated with chlamydial infections, further studies using more accurate tests are needed to fully assess the burden of chlamydial coinfection with malaria and thus, provide details evidence for decision making.

We noted that first pregnancy women were at higher risk of malaria and STI/RTI coinfections. The high susceptibility of primigravid women to malaria [10], combined with the high prevalence of STI/RTI reported in the current study increased the risk of malaria and STI/RTI coinfection. Therefore, it would be more effective to systematically screen and treat this population or explore alternative combination drug that can prevent malaria infection and adequately treat STI/RTI without the need of diagnostic. The intermittent preventive treatment of malaria in pregnancy using sulfadoxine–pyrimethamine is already available for malaria control in pregnancy. Azithromycin regimens are widely used for the treatment of STI/RTI in pregnancy, thus would be a feasible alternative in association with the IPTp-SP in the study settings [30, 31].

As expected in this study setting, there was no direct link between clinical symptoms and coinfection. Indeed, only 10% of cases were symptomatic, and yet nearly 90% would have been missed and not adequately managed. This confirms that clinical symptoms cannot reliably predict neither malaria infection [11] nor STI/RTI [9] in pregnant women. Hence, exploring alternative method is needed. The difficulties of STI/RTI diagnostic due to the absence of symptoms combined with the weaknesses of local laboratory infrastructures in rural area makes the approach of screen and treat less feasible. Thus, exploring a drug that can be combined to the IPTp-SP to adequately treat STI/RTI is needed. Azithromycin is efficacious for the treatment of chlamydial infection [32], syphilis [33, 34] and other bacterial causes of preterm birth [35]. This drug was also tested for the intermittent preventive treatment of malaria in pregnancy in which it had shown comparable result to the IPTp-SP in reducing adverse birth outcomes [36, 37]. Thus, combining these two drugs through a systematic administration approach could substantially reduce malaria and STI/RTI coinfections and their deleterious effects on birth outcomes. Data are needed to evaluate the feasibility and efficacy of this approach in pregnant women in resource-limited settings.

In this study, we described malaria and curable sexually transmitted/reproductive tract coinfection in pregnant women in a rural health district of Burkina Faso. Some limitations are worth noting. Important infections such as gonorrhea were not tested due to the absence of rapid test or an easy to implement laboratory diagnostic method and this understated the actual magnitude of coinfection in the study population. In addition, the blood smear microscopy-based diagnosis of pregnancy malaria might have understated the prevalence of malaria and STI/RTI coinfection in the current study through its relative limited performances. Gestational age was collected using the mother’s knowledge of last menstrual period or the obstetrical examination in majority of cases due to the absence of ultrasounds, and this process is frequently prone to errors. However, the study is worth emphasizing the burden of malaria and STI/RTI coinfections and suggest that improved approaches are needed.

## Conclusion

In this study population, the prevalence of malaria and sexually transmitted/reproductive tract coinfections were high and were not predicted by the clinical symptoms. Thus, exploring an alternative approach to systematically prevent malaria and treat STI/RTI would be of public health interest.

## Data Availability

The data sets used and/or analyzed during the current study are available from the corresponding author on reasonable request.

## Abbreviations

STI/RTI: Sexually transmitted/reproductive tract infections
SP: Sulfadoxine–pyrimethamine
IPTp: Intermittent preventive treatment of malaria in pregnancy
ANC: Antenatal care
OR: Odds ratio
aOR: Adjusted odds ratio
CI: Confident interval
WHO: World health organization
LMP: Last menstrual period
CRUN: Clinical Research Unit of Nanoro
